# GENDER DIFFERENCES IN THE MECHANISM OF INVOLUNTARY RETIREMENT ON LONELINESS

**DOI:** 10.1093/geroni/igz038.1958

**Published:** 2019-11-08

**Authors:** Oejin Shin, hyunjoo Lee, Sojung Park, Ji Young Kang

**Affiliations:** 1 University of Illinois at Urbana Champaign, Urbana Champaign, Illinois, United States; 2 Daegu University, Gyeongsan-si, Gyeongsangbuk-do, Korea, Republic of; 3 Brown School, Washington University in St. Louis, St. Louis, Missouri, United States; 4 Hannam University, Daejeon, South Korea, Korea, Republic of

## Abstract

Involuntary retirement is known to be associated with long-lasting negative effects on well-being compared to voluntary retirement. However, little is known about complex mechanism connecting the path from social contexts and psychological factors of retirees, especially involuntary retirees to later year well-being. Also, despite the well-known gendered pattern of preretirement employment histories over the life course in general, gender differences in the pathway on well-being after involuntary retirement is still unclear. Drawing on the stress process theory, this study examined gender difference on the pathway linking involuntary retirement (primary stressor) to loneliness through material/physical vulnerability (secondary stressor) and social support/self-efficacy (coping resources). Data are from the 2014 HRS with 2,087 retirees aged 65+. Two-step structural equation modeling (SEM) was utilized to examine the significance of the specific effects of multiple mediators (material/physical vulnerability, coping resources). For male retirees, involuntary retirement was associated with a higher level of loneliness mediated through physical vulnerability and social-efficacy. For female retirees, involuntary retirement was directly associated with loneliness as well as indirectly associated through 1) material vulnerability connected to low social support, and 2) physical vulnerability related to low social support and low social-efficacy. The different impact of involuntary retirement may be due to differences in work history, previous work quality, and accumulated financial condition across gender. The results suggest important gender specified implications for social policy and practice for involuntary retirees.

